# Mitochondrial redox system: A key target of antioxidant therapy to prevent acquired sensorineural hearing loss

**DOI:** 10.3389/fphar.2023.1176881

**Published:** 2023-03-31

**Authors:** Jeong-In Baek, Ye-Ri Kim, Kyu-Yup Lee, Un-Kyung Kim

**Affiliations:** ^1^ Department of Companion Animal Health, College of Rehabilitation and Health, Daegu Haany University, Gyeongsan, Republic of Korea; ^2^ Department of Biology, College of Natural Sciences, Kyungpook National University, Daegu, Republic of Korea; ^3^ Advanced Bio-Resource Research Center, Kyungpook National University, Daegu, Republic of Korea; ^4^ Department of Otorhinolaryngology-Head and Neck Surgery, School of Medicine, Kyungpook National University, Daegu, Republic of Korea; ^5^ School of Life Sciences, KNU Creative BioResearch Group (BK21 Plus Project), Kyungpook National University, Daegu, Republic of Korea

**Keywords:** acquired hearing loss, noise, ototoxic drugs, ROS, mitochondria, drug development

## Abstract

Noise (noise-induced hearing loss), and ototoxic drugs (drug-induced ototoxicity), and aging (age-related hearing loss) are the major environmental factors that lead to acquired sensorineural hearing loss. So far, there have been numerous efforts to develop protective or therapeutic agents for acquired hearing loss by investigating the pathological mechanisms of each types of hearing loss, especially in cochlear hair cells and auditory nerves. Although there is still a lack of information on the underlying mechanisms of redox homeostasis and molecular redox networks in hair cells, an imbalance in mitochondrial reactive oxygen species (ROS) levels that enhance oxidative stress has been suggested as a key pathological factor eventually causing acquired sensorineural hearing loss. Thus, various types of antioxidants have been investigated for their abilities to support auditory cells in maintenance of the hearing function against ototoxic stimuli. In this review, we will discuss the scientific possibility of developing drugs that target particular key elements of the mitochondrial redox network in prevention or treatment of noise- and ototoxic drug-induced hearing loss.

## 1 Introduction

Sensorineural hearing loss (SNHL), the most common type of permanent hearing impairment, is caused by physical and/or functional damage of cochlear hair cells in inner ear or auditory nerves including spiral ganglion neurons. Acquired SNHL, resulting from continuous accumulation of cellular damage due to various environmental stimuli, increases in prevalence with aging: Being identified in approximately 5–8 of 1,000 children, 33% of adults 65–74 years of age, and over 80% of adults 85 years of age (Walling et al., 2012; Baumgartner et al., 2021). This prevalence of acquired SNHL is much higher than congenital SNHL that occurs in two to four of 1,000 newborns. The major environmental factors that lead to acquired SNHL are aging (age-related hearing loss), noise (noise-induced hearing loss), and ototoxic drugs (drug-induced ototoxicity). Over the past few decades, there have been numerous efforts to develop therapeutic agents for each type of acquired hearing loss by investigating the pathological mechanisms of hearing loss at the molecular level. An imbalance in mitochondrial reactive oxygen species (ROS) levels that enhance oxidative stress has been suggested as a key pathological factor that eventually causes hair cell death in all three types of acquired SNHL (Bottger and Schacht, 2013; Fujimoto and Yamasoba, 2014; Kamogashira et al., 2015; Wong and Ryan, 2015). Attempts to decrease ROS levels in order to prevent or slow acquired SNHL led researchers to test the protective or alleviative effects of various antioxidants, such as vitamins, lipoic acids, polyphenols, and other small molecules (Hildebrand et al., 2008; Tavanai and Mohammadkhani, 2017; Ibrahim et al., 2018; Fetoni et al., 2019; Pak et al., 2020). Most of these investigations have been conducted in animal models with only a few studies in humans, and there is still a lack of information on the underlying mechanisms of redox homeostasis and molecular redox networks in hair cells. Due to these limitations, there is currently only one medicine at a clinically applicable level. In 2022, the Food and Drug Administration (FDA) of United States approved the use of sodium thiosulfate, an antioxidant, as a therapeutic agent of cisplatin-induced hearing loss, based on the results of clinical trials (Freyer et al., 2017; Brock et al., 2018a; Brock et al., 2018b). In the clinical test, sodium thiosulfate successfully reduced the incidence of cisplatin-induced ototoxicity by nearly 50% in the hepatoblastoma patients. However, verification of the therapeutic effect was limited to patients under the age of 18 with non-metastic solid cancer, and the effect has also been shown to vary depending on the time intervals between administration of cisplatin and sodium thiosulfate (Hazlitt et al., 2018). It means, the development of otoprotectants that are effective for a wide range of acquired hearing loss is still needed. In this review, we will discuss previous efforts to develop protective or therapeutic agents focusing on oxidative stress-induced mitochondrial damage in hair cells. We will also propose the possibility of developing drugs that target particular elements of the mitochondrial redox network in hearing loss pathology.

## 2 Oxidative stress in acquired sensorineural hearing loss

**FIGURE 1:**
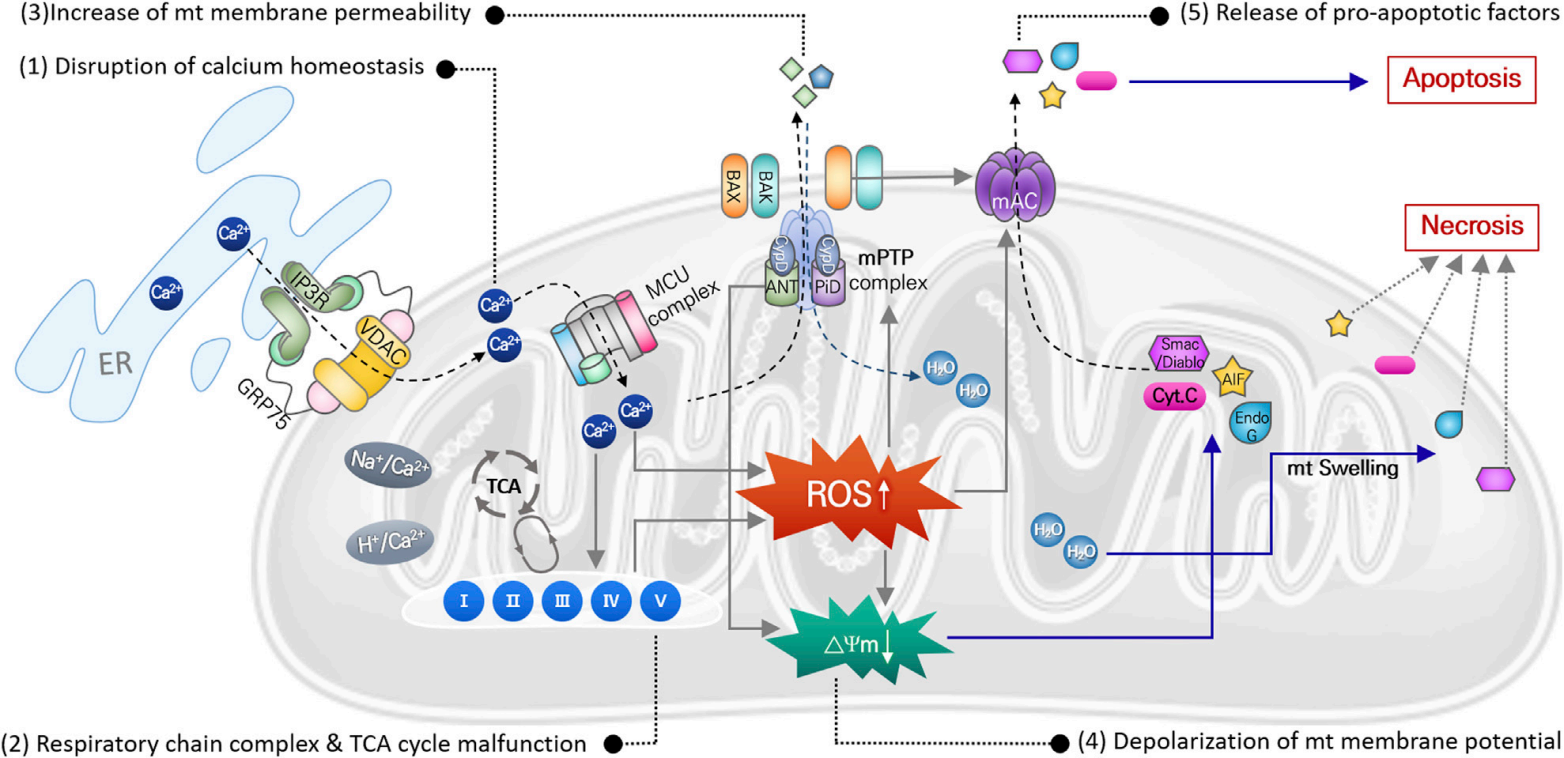
Noise, aging and ototoxic drug-induced intracellular signaling pathways leading to hair cell death. It indicates that organelle stress (ER and mitochondria), inflammation, MAPK signaling pathways induced by three environmental stimuli are all mutually interact each other, which finally leads to apoptosis or necroptosis of hair cells. Importantly, increase in mitochondrial ROS is at the center of all the pathways.

The main pathological factors of acquired SNHL, aging, noise exposure, and ototoxic drugs, induce multiple, simultaneous responses in cochlear hair cells, that directly damage macromolecules (nucleic acids, proteins, and lipids), change ion homeostasis, and activate/inhibit intrinsic and extrinsic signaling pathways (Wong and Ryan, 2015; Wu et al., 2020). These responses ultimately produce irreversible hair cell damage when the mechanical or biochemical stimuli overwhelm cellular homeostatic capacity. Disruption of cellular redox homeostasis by either or both increased ROS generation and inhibition of antioxidant defense systems, is known to be mutually influenced by other cellular responses induced by noise, drugs, and aging (Wu et al., 2020) (Figure 1).

### 2.1 Noise-induced oxidative stress and alleviative effects of antioxidants

Intense noise can directly cause mechanical disruption of the hair cell stereocilia, resulting in their dysfunction in the auditory pathway (Slepecky, 1986; Patuzzi et al., 1989). However, most noise-induced hearing loss is caused by an accumulation of biochemical damage due to prolonged exposure to sound stimuli below the threshold of mechanical damage. Stimulation of the mitochondrial respiratory system by ischemia/reperfusion (Henderson et al., 2006) and release of free Ca^2+^ from Ca^2+^ stores, such as endoplasmic reticulum (ER) to cytosol and into mitochondria (Gorlach et al., 2015; Wong and Ryan, 2015), are the major noise-induced intracellular responses that cause biochemical hair cell damage. Importantly, these processes are commonly linked to overgeneration of ROS (Yamashita et al., 2004a). Noise exposure also induces insufficiency of the antioxidant system. Consequently, increased ROS randomly reacts with lipids, nucleic acids, and proteins, resulting in dysfunction of these macromolecules, and subsequently inducing release of pro-apoptotic factors. Cytochrome C promotes caspase 3-mediated apoptosis, and translocation of Endonuclease G (EndoG) and apoptosis-inducing factor (AIF) from mitochondria into the nucleus, triggering apoptotic hair cell death with condensed nuclei (Yamashita et al., 2004b; Wong and Ryan, 2015; Sha and Schacht, 2017). Increased ROS has also been shown to activate the c-Jun N-terminal kinase (JNK) signaling pathway, leading to apoptosis in an animal model (Wang et al., 2007; Wu et al., 2020).

Because oxidative stress has been strongly implicated as a cause of noise-induced hearing loss, various types of antioxidant molecules have been used to protect or recover hair cells. Glutathione, D-methionine, resveratrol, salicylate, ebselen, and coenzymeQ_10_ administered in animal studies had significant protective effects on noise-induced hair cell damage (Sha and Schacht, 2017; Pak et al., 2020). Specifically, N-acetyl cysteine (NAC), *α*-lipoic acid, and ebselen have advanced to clinical trials and shown a substantial otoprotective effect. In two randomized clinical studies, a group of textile workers who received NAC had a reduced temporary threshold shift after noise exposure (Fetoni et al., 2009), as well as army members who received NAC for 14 days who had a reduced temporary threshold shift (Lorito et al., 2008; Kopke et al., 2015). Alpha-lipoic acid also had a protective effect on noise-induced hearing threshold shift. When a group of healthy subjects received oral *α*-lipoic acid 1 h before noise exposure, their temporary threshold shift at 6 kHz was reduced after noise exposure (Campbell et al., 2007). These results suggest a strong possibility that reducing ROS accumulation may effectively prevent noise-induced hearing loss.

### 2.2 ROS accumulation in drug-induced ototoxicity and therapeutic effects of antioxidants

Aminoglycoside, broad-spectrum antibiotics, and platinum-based anticancer agents such as cisplatin are the most well-known ototoxic drugs that can cause irreversible, bilateral, and high frequency hearing loss (Musial-Bright et al., 2011). Following entry into the hair cells, both aminoglycosides and cisplatin directly bind to hundreds of intracellular proteins such as kinases, transcription factors, and ion channels that potentially cause dell death (Karasawa et al., 2010; Karasawa et al., 2011; Karasawa et al., 2013; Karasawa and Steyger, 2015).

Although they affect multiple cellular signaling pathways by directly interacting with various proteins, the central affected pathological pathway is the accumulation of oxidative stress caused by accelerated ROS generation and inhibition of antioxidant enzyme activities, eventually leading to apoptosis or necrosis of hair cells (Steyger, 2021). Aminoglycosides are known to accelerate both enzymatic and non-enzymatic ROS formation (Priuska and Schacht, 1995), thereby activating the JNK signaling pathway followed by apoptotic hair cell death (Mielke and Herdegen, 2000). In enzymatic ROS formation, nicotinamide adenine dinucleotide phosphate (NADPH) oxidases are considered a primary source of ROS generation. NADPH oxidase is a protein complex composed of several subunits including a catalytic subunit, NOX (Altenhofer et al., 2015; Steyger, 2021). In previous studies, NOX2 expression was abundantly increased in outer hair cells after neomycin administration in rat cochlea, and inhibition of NOX2 significantly reduced neomycin-induced hair cell damage (Qi et al., 2018). Cisplatin also upregulates NOX3 expression and activates the NOX3 signaling pathway, increasing superoxide production in cultured cells and rat cochlea (Banfi et al., 2004; Mukherjea et al., 2008; Mukherjea et al., 2011).

Disruption of the intracellular antioxidant system also contributes to aminoglycoside- or cisplatin-induced oxidative stress. For example, cisplatin can bind directly to sulfhydryl groups within antioxidant enzymes causing enzyme dysfunction, and can also deplete glutathione (GSH) and NADPH that are essential factors for antioxidant enzyme function (Rybak et al., 2007). Cisplatin and kanamycin are known to decrease expression and activity of the primary antioxidant enzymes: Superoxide dismutase (SOD), glutathione peroxidase (GPx), glutathione reductase (GR), and catalase (CAT) (Rybak et al., 2000; Rybak et al., 2007). Although there are various molecular factors directly affected by drugs, ultimately they cause an excessive accumulation of ROS, and numerous studies have shown that the subsequent cell death signaling pathways shared with noise-induced hearing loss.

Various types of antioxidants were also tested in drug-induced ototoxicity to examine their ability to prevent hearing loss. The widely-used antioxidants, NAC, sodium thiosulfate, and D-methionine, effectively protected or rescued cisplatin-induced ototoxicity, due to their high affinity for platinum molecules, as well as their antioxidative activity (Wu et al., 2020). In our previous studies, we identified several antioxidants that had protective effects against drug-induced ototoxicity. In mouse cochlear explants treated with amikacin, kanamycin, or cisplatin, pre-treatment with galangin (flavonoid antioxidant), fursultiamine (thiamine disulfide derivative), or berberine chloride (alkaline isoquinoline) reduced intracellular ROS levels in hair cells. These treatments prevented ROS-mediated caspase-3 activation, DNA fragmentation, and apoptosis of hair cells, indicating that an antioxidant can be used to prevent both noise- and drug-induced hearing loss. Sodium thiosulfate (STS) is currently the only clinically approved treatment for cisplatin-induced ototoxicity. As an antioxidant, it is known to plays a role in directly removing ROS and promote antioxidative and anti-apoptotic enzymes by activating Nuclear factor erythroid-related factor 2 (Nrf2) (Zhang et al., 2021). Moreover, it directly binds to cisplatin, inhibiting and deactivating the metabolism of cisplatin by formation of platinum (Pt)-STS complex. Cisplatin is metabolized and activated in the body through the process of hydrolysis. In this process, each of activated cisplatin metabolites has different tumor selectivity, finally causing normal cell damages. Since reducing the level of activated form of cisplatin inhibit cytotoxicity of normal cells, it is suggested that the amelioration of cisplatin-induced cytotoxicity by STS could be explained in terms of the rapid formation of inactive Pt-STS complex (Sooriyaarachchi et al., 2012). Therefore, it is thought that STS can play a role as a direct inhibitor of cisplatin, selectively reducing toxicity to normal cells, rather than as an antioxidant.

## 3 Mitochondrial dysfunction caused by ROS in acquired hearing loss

### 3.1 Oxidative stress-induced mitochondrial damage triggers hair cell death signals

**FIGURE 2:**
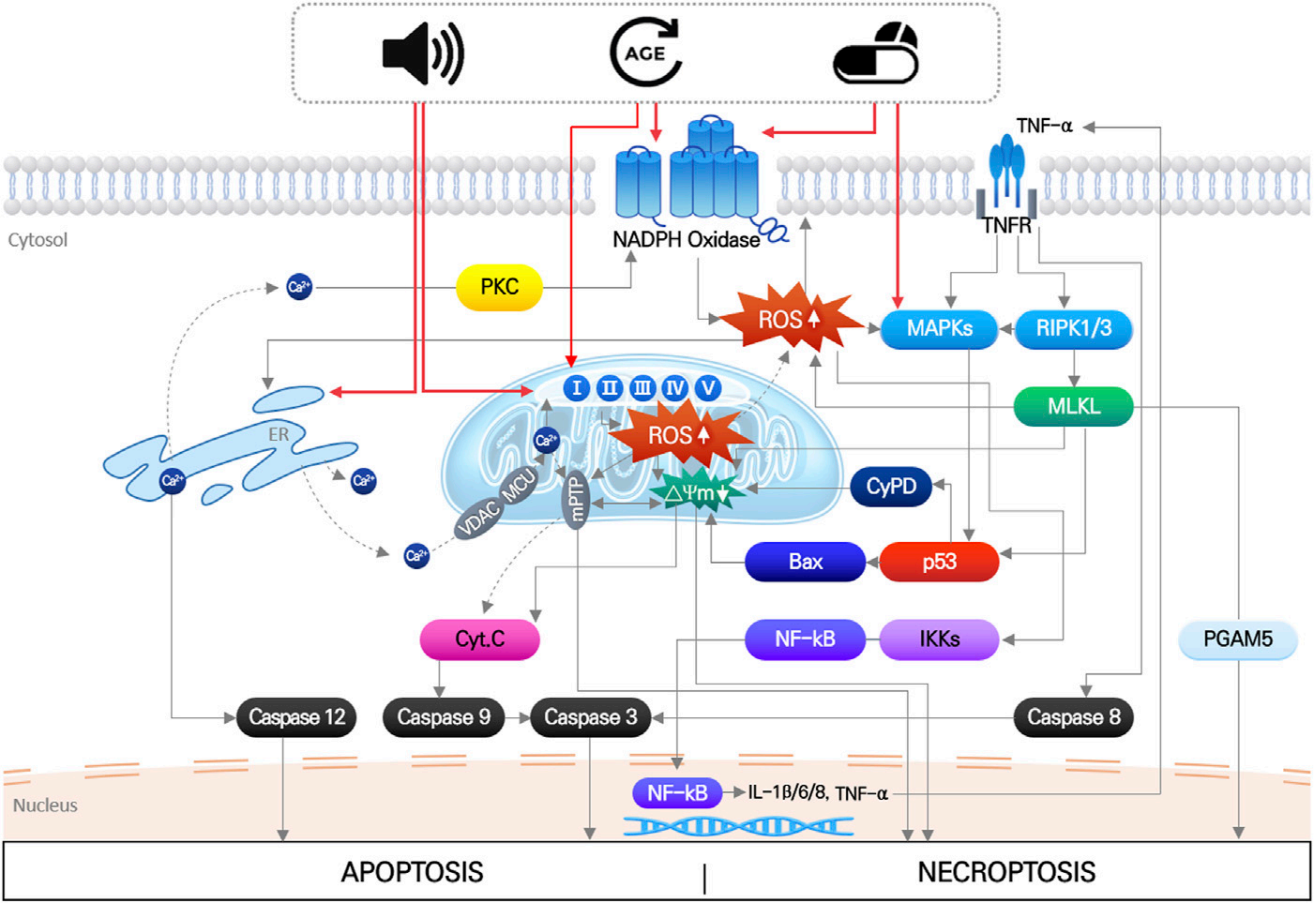
Mitochondrial ROS-induced damages causing mitochondrial dysfunction. Direct damages of mitochondrial DNA and antioxidant enzyme, or disruption of ion homeostasis 1), or malfunction of the mitochondrial respiration system 2), can cause increase in mitochondrial ROS. It subsequently leads to increase in mitochondrial membrane potential 3) with depolarization of membrane potential 4), causing osmotic swelling of mitochondria and release of pro-apoptotic factors 5). Finally, cell death signaling pathways are activated by these apoptotic factors.

Mitochondria, referred to as the center of cellular energy metabolism, are organelles that synthesize ATP through the TCA cycle and oxidative phosphorylation processes. Because this aerobic respiration unavoidably produces ROS, mitochondria are the primary cellular source of ROS (Wei et al., 2001; Islam, 2017). Mitochondria also play critical roles in maintaining cellular homeostasis and contribute to homeostatic regulation of calcium and iron concentrations (Contreras et al., 2010), autophagy (Scherz-Shouval and Elazar, 2007), and cell death (Borutaite, 2010). Thus, mitochondrial damage or dysfunction can directly activate cell death signaling pathways. Noise- or cytotoxic drug-induced intracellular responses, including ischemia/reperfusion, DNA damage, ER stress, etc., increase ROS levels and DNA damage in mitochondria within auditory cells. Even though mitochondrial ROS can be easily scavenged by mitochondrial antioxidant systems under normal physiological conditions, excessive ROS generation or failure to remove mitochondrial ROS can cause oxidative stress leading to mitochondrial dysfunction in multiple ways (Figure 2).

In mitochondria, free Ca^2+^ ions are the most likely to trigger ROS elevation. Particularly after intense noise stimulation, Ca^2+^ is released from the ER and translocated into mitochondria through an ion transporter such as VDAC or MCU. Abnormal Ca^2+^ influx can dysregulate Ca^2+^-regulated enzymes that are involved in ROS metabolism, leading to increased mitochondrial ROS levels, free-radical damage, and ultimately to mitochondrial dysfunction (Tretter and Adam-Vizi, 2004; Bottger and Schacht, 2013). Accumulated mitochondrial ROS can directly damage mitochondrial DNA (mtDNA), causing mtDNA mutations. Accumulation of mtDNA is responsible for dysfunction of various mitochondrial proteins, which disrupts metabolic homeostasis and induces mitochondrial dysfunction (Baker and Staecker, 2012). Finally, mitochondrial damage caused by increased mitochondrial ROS can depolarize mitochondrial membrane potential, which increases mitochondrial membrane permeability and initiates apoptosis or necrosis signaling pathways. Increased ROS activates p53 in the cytosol, leading to translocation of Bcl-2 associated X (BAX) from the cytosol to the mitochondrial outer membrane. Mitochondrial ROS-induced activation of the BAX pore and other mitochondrial pore complexes, e.g., the mitochondrial permeability transition pore (mPTP) and mitochondrial apoptosis-induced channel (mAC), contributes to the release of pro-apoptotic factors (cytochrome C, EndoG, and AIF) from the mitochondrial matrix to the cytosol to activate the apoptotic signaling pathway (Dejean et al., 2005; Briston et al., 2017). By contrast, loss of mitochondrial membrane potential activates the mPTP pore complex, allowing H_2_O and other small molecules to enter the mitochondrial matrix, which induces mitochondrial osmotic swelling and initiates the necrosis signaling pathway (Briston et al., 2017).

Aminoglycosides tend to accumulate in the hair cell mitochondria, and oxidative stress-induced mitochondrial damage and subsequent cell death by antibiotics are also observed consistently in cochlear hair cells (Hobbie et al., 2008). Gentamicin directly inhibits mitochondrial protein synthesis, which triggers opening of the mPTP pore complex that can then release pro-apoptotic factors (Dehne et al., 2002). Cisplatin administration increases mitochondrial ROS, leading to loss of mitochondrial membrane potential and BAX expression, which causes increased cleaved caspase-3 expression and hair cell apoptosis in mouse cochlear explants (Kim et al., 2018).

### 3.2 Prevention of hair cell death through intensive targeting of mitochondrial ROS

Because mitochondria act as a pathological link between intracellular oxidative stress and apoptotic cell death, inhibiting mitochondrial oxidative stress or decreasing mitochondrial membrane permeability using mitochondria-targeted molecules, could be an effective approach to alleviating noise- and drug-induced hearing loss. Thus, mitochondria-specific targeting of potent compounds has been considered important to maximize effectiveness of the compounds. Especially Lipophilic cation-based modification of compounds is one of the most successful mitochondria-targeting techniques, due to improved ability to cross polarized mitochondrial membrane. Triphenylphosphonium (TPP), a kind of lipophilic cation, has been conjugated to various potent antioxidants (Wang et al., 2020), and numerous TPP-tagged mitochondria-targeted antioxidants including MitoQ, SkQ1, SkQR1, MitoVitE, and MitoPeroxidase, have been tested to prevent or alleviate acquired hearing loss (Zielonka et al., 2017; Fujimoto and Yamasoba, 2019).

We previously used a mitochondria-targeted antioxidant, MitoQ, to evaluate the therapeutic potential of mitochondria-specific antioxidants in oxidative stress-induced hair cell damage. In mouse cochlear explants, H_2_O_2_ treatment increased mitochondrial ROS leading to loss of mitochondrial membrane potential, which was attributed to decreased expression of the mitochondrial respiratory chain complex I, III, and V. When MitoQ was administered 1 h prior to H_2_O_2_ treatment, the mitochondrial oxidative stress responses were almost completely neutralized, thus protecting hair cells from apoptotic cell death (Kim et al., 2019). Other studies on drug-induced ototoxicity also suggested that both MitoQ and SkQR1 significantly reduce aminoglycoside- and cisplatin-induced hearing loss in cultured cells and animal models (Jankauskas et al., 2012; Ojano-Dirain and Antonelli, 2012; Ojano-Dirain et al., 2014; Tate et al., 2017; Dirain et al., 2018).

## 4 Conclusion

Extensive scientific evidence strongly suggests mitochondria as a key target to protect auditory cells and maintain hearing function. Although a number of previous studies have shown that general antioxidants can provide protection against drug-induced ototoxicity, there are two significant limitations. First, since the general antioxidants are not highly selective for specific target molecules and can affect multiple signaling pathways simultaneously with low specificity, it is difficult to determine drug efficacy and safety at low concentrations. Second, general antioxidants have the potential to cause unintended side effects when co-administered with other medicines. For instance, antioxidants such as *α*-lipoic acid can inhibit the death of cancer cells as well as normal cells, thereby inhibiting the anticancer effect. Thus, *α*-lipoic acid cannot be administered in combination with the anticancer drugs such as cisplatin.

To overcome these limitations, it will be important to discover and develop novel therapeutic agents that interact with specific mitochondrial factors that contribute to mitochondrial redox homeostasis, such as antioxidant enzymes or mitochondrial pore proteins. This will be essential information for developing common drugs that are widely effective in different types of acquired hearing loss.
